# Biomimetic Curcumin-Loaded Liposomes for the Treatment of Dry Eyes and Meibomian Gland Dysfunction: An In Vivo Study

**DOI:** 10.3390/jcm13216436

**Published:** 2024-10-27

**Authors:** Vytautas Baranauskas, Ruta Jaruseviciene, Mantas Grigalavicius, Saulius Galgauskas, Vitalijus Karabanovas, Simona Steponkiene

**Affiliations:** 1 Institute of Biochemistry, Life Science Centre, Vilnius University, Sauletekio av 7, LT 10257 Vilnius, Lithuania; 2 Faculty of Medicine, Vilnius University, M. K. Ciurlionio Str. 21, LT 03101 Vilnius, Lithuaniasaulius.galgauskas@mf.vu.lt (S.G.); 3 Laser Research Center, Faculty of Physics, Vilnius University, Saulėtekio av. 9, III bld., LT 10222 Vilnius, Lithuania; mantas.grigalavicius@cr.vu.lt; 4 Biomedical Physics Laboratory, National Cancer Institute, Santariskių Str. 1, LT 08660 Vilnius, Lithuania; vitalijus.karabanovas@nvi.lt; 5 Department of Chemistry and Bioengineering, Vilnius Gediminas Technical University, Saulėtekio al. 11, LT 10223 Vilnius, Lithuania

**Keywords:** liposome, dry eye syndrome, meibomian gland dysfunction, cyclosporine A, curcumin, in vivo

## Abstract

**Background/Objectives:** Meibomian gland dysfunction (MGD) and dry eye syndrome (DES) are common eye diseases characterized by altered tear film stability and inflammation of the ocular surface, causing significant discomfort and possible visual impairment. This study aimed to investigate the efficacy of curcumin-loaded liposomes (Lipo@Cur) compared to cyclosporine A-loaded liposomes (Lipo@CycA) in experimental rabbit models of MGD and DES, with a focus on their ability to improve tear film stability and reduce ocular surface inflammation. **Methods:** MGD and DES were induced using complete Freund’s adjuvant (CFA) and treated to evaluate the effect of liposomal formulations on tear break-up time (TBUT), clinical signs of inflammation (telangiectasia, conjunctival hyperemia, meibomian foramen occlusion), and corneal as well as conjunctival histological cells. **Results:** Lipo@Cur increased TBUT and reduced the signs of ocular surface inflammation, potentially approaching the effectiveness of clinically approved cyclosporine A encapsulated in liposomes (Lipo@CycA). Histological analysis suggested improvements in corneal epithelial thickness and goblet cell density in the treated groups, which may indicate a reversal of DES-induced damage to the ocular surface. **Conclusions:** Plant-originated curcumin encapsulated in liposomes offers a promising therapeutic strategy for the management of MGD and DES that may improve patient outcomes by addressing the underlying inflammatory mechanisms of these conditions.

## 1. Introduction

Dry eye syndrome (DES) is a common chronic disorder that affects millions of people around the world. The prevalence of the disease fluctuates from 5 to 50 percent of the world population [1]. The number of cases is likely to increase due to an aging population. According to the World Health Organization (WHO), by 2050, the world’s population of people aged 60 years and older will double to 2.1 billion [2].

There are two main types of DES: aqueous deficiency (tear deficiency) and evaporative. They can also coexist [3]. The tear film has three layers: the outer lipid layer, the middle aqueous layer, and the inner mucin layer, which interacts with the corneal epithelial surface [4]. The outer lipid layer of the tear film plays an important role in inhibiting tear film evaporation [5]. Meibomian gland dysfunction (MGD) is the main reason for the disruption of the outer lipid layer. Without treatment, MGD leads to DES [6]. The main treatment for mild DES is artificial tears. In more severe cases, the treatment requires cyclosporine A, corticosteroids, mucosecretory drugs, antibiotics, or nonsteroidal anti-inflammatory drugs.

Ocular drug absorption is complicated due to the protective mechanisms and natural barriers of the eye; therefore, achievement of an optimum drug concentration within the target site is still a problem. The most common administration method of ocular drugs is through the topical route as eye drops. After local application, the drug must be absorbed either through the corneal route (cornea, aqueous humor, intraocular tissues) or the non-corneal route (conjunctiva, sclera, choroid/retinal pigment epithelium) [7]. In order to develop a suitable delivery carrier, it is necessary to understand all the barriers of the eye. The cornea is divided into six different cell layers: epithelium, Bowman’s layer, stroma, Dua’s layer, Descemet’s membrane, and endothelium [8]. Each layer offers a different polarity and potential obstacle for drug permeation. Common ophthalmic drug formulations, such as solutions and suspensions, have poor bioavailability [9]. Nanomaterials have been explored as innovative as drug delivery systems to improve conventional drugs [10]. Such formulations as nanoparticles, liposomes, dendrimers, and niosomes have been developed to increase drug bioavailability and reduce adverse effects [11].

One of the most promising delivery systems for DES is drug-loaded liposomes [12]. It helps to obtain a better treatment effect and reduces the need for frequent moisturizing, leading to better patient satisfaction [13]. Structurally, liposomes are spherical or multilayered spherical vesicles made of physiologically acceptable natural or synthetic phospholipids [14]. Liposomes also have excellent properties of biocompatibility, feasibility, and tenability [12]. Liposomes as drug delivery systems have the ability to increase the solubility of hydrophilic or hydrophobic drugs. Therefore, liposomes may be useful in other forms of therapy. Comparing other ophthalmic drug delivery carriers and drug-loaded liposomes, the latter exhibit improved tolerance, drug delivery and penetration, and corneal healing in rabbit models [15,16].

Cyclosporine A is a routinely prescribed drug for patients with chronic DES, and 0.05% ophthalmic emulsion is available at pharmacies. It is in a class of medications called immunomodulators. Cyclosporine A is a neutrally charged lipophilic molecule with low water solubility, which causes challenges for the development of a safe and effective ocular drug delivery system. Cyclosporine A reduces inflammation associated with DES and thus improves tear production. Cyclosporine A ophthalmic solutions were originally formulated in oil-based solvents, such as castor oil or corn oil, but they caused side effects, including blurred vision, burning, and stinging, and were poorly tolerated by patients [17]. Due to the positive effect of cyclosporine A for DES treatment, scientists are still seeking to find the best way for its administration and drug delivery. Curcumin is the active ingredient in the herbal remedy and dietary spice turmeric (Curcuma longa Linn). It has been used for medicinal purposes for thousands of years in traditional Chinese medicine and Ayurvedic medicine in Asian countries. Curcumin is consumed as an active ingredient in herbal remedies to treat liver diseases, rheumatological diseases, diabetes, atherosclerosis, infectious diseases, and cancer [18]. It has antioxidant, anti-inflammatory, antimutagenic, antimicrobial, and anticancer activities. However, curcumin has poor absorption as well as rapid metabolism and elimination. Various nanomaterials have been employed to overcome the solubility and stability challenges of curcumin [19]. Encapsulation of curcumin into liposomes could increase its bioavailability, solubility, and stability [20]. Positive clinical and preclinical investigations suggest that curcumin may be used for several ocular diseases, such as chronic anterior uveitis, diabetic retinopathy, glaucoma, age-related macular degeneration, and DES. These studies used oral curcumin alone or curcumin in phytosomes made of phospholipids in order to protect it from intestinal hydrolysis, which increases the bioavailability by 20 times compared with plain curcumin [21]. Although curcumin has a scientifically proven anti-inflammatory profile, studies on the anti-inflammatory action of curcumin applied topically within the cornea and periocular region are still missing.

**Figure 1:**
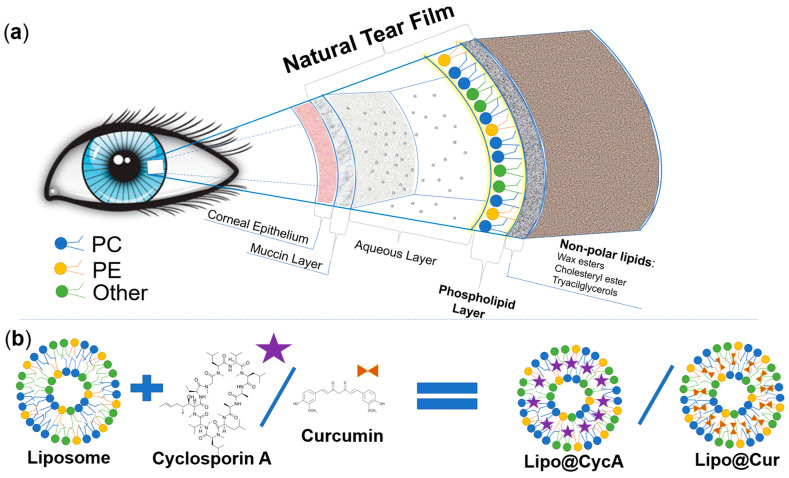
Schematic representation of the different constituents of the natural tear film (**a**) and comparison between lipids of the natural tear film and the lipids of Lipo@Cur/Lipo@CycA (**b**). Colors of phospholipids in section (**a**) and section (**b**) represent these phospholipids: **blue**—phosphatidylcholine (PC), **yellow**—phosphatidylethanolamine (PE), and **green**—other phospholipids (Other). Matching colors emphasizes the similarities between natural tear film and phospholipid coating of Lipo@Cur/Lipo@CycA.

This study aimed to develop biomimetic anti-inflammatory eye drops for the treatment of MGD and DES. Before designing a new ocular formulation for MGD and DES, we analyzed the physiology of the eye and the physicochemical properties of the different components of the natural tear film of the eye. Figure 1a was drawn according to the physiology of tear film [4,22,23]. Greiner et al. investigated the exact composition of tear film lipids by NMR spectroscopy [24]. In our study, we chose phospholipids that are similar to the ones naturally produced by the meibomian glands. The natural meibomian gland phospholipids, estimated by Greiner et al., and the ones used for the production of drug-loaded liposomes were compared (Table 1). We aimed to mimic characteristics of the natural tear film; therefore, the main lipids that were chosen were phosphatidylcholine, phosphatidylethanolamine, and phosphatidylinositol with a molar ratio similar to the natural tear film (Table 1). Two hydrophobic anti-inflammatory substances, cyclosporine A and curcumin, were encapsulated into biomimetic liposomes (Figure 1b). Cyclosporine A is a clinically approved compound for the treatment of DES [4], while curcumin’s efficacy in the treatment of DES or MGD has never been tested before.

The efficacy of the treatment was evaluated by the Schirmer test, tear break-up time (TBUT), and clinical assessment of the eyelids in a rabbit model. We hypothesized that a formulation combining mixed phospholipids and anti-inflammatory substances, cyclosporine A or curcumin, could synergistically reduce the inflammation of meibomian glands and replenish the destabilized tear film. As a positive indication of a successful treatment, the symptoms of DES and MGD decreased.

## 2. Materials and Methods

### 2.1. Reagents

L-α-Phosphatidylcholine (Soy-40%) containing 40% phosphatidylcholine (PC) purified from soy lecithin, cyclosporine A (purity > 98.5%), curcumin (purity > 99.6%), and protamine sulfate were purchased from Sigma-Aldrich (Darmstadt, Germany). Sodium chloride (0.9% sterile solution) was obtained from the local pharmacy. Rotisolv^®^ hplc gradient-grade acetonitrile and water were purchased from ROTH (Carl Roth, Karlsruhe, Germany). According to Sigma-Aldrich manufacturer, 100 mg of Soy-40% contains 40 mg phosphatidylcholine (PC), 16 mg phosphatidylethanolamine (PE), and 11 mg phosphatidylinositol (PI).

### 2.2. Preparation of Liposomes by Thin-Film Hydration

Liposomes were prepared according to the technique described by Elsana et al. [25] and modified by our research group. Briefly, Soy-40% and curcumin or cyclosporine A (25:1 mg/mL) were dissolved in chloroform, and the organic solvent was slowly removed using a rotating round bottom flask immersed in a water bath at 47 °C. Argon gas stream was used to speed up the evaporation. A thin film of dry lipids together with the active substances (curcumin or cyclosporine A, accordingly) formed on the inner surface of the flask and was hydrated with an aqueous solution of 0.123 M NaCl at 47 °C for 1 h. Empty liposomes were prepared according to the same procedure written above, except no active substances were added. The names and corresponding formulations of liposomal eye drops are given in Table 2.

To ensure sterility, the ophthalmic formulation was prepared under aseptic conditions. All glassware and plastic tubes were sterilized by dry heat at 100 °C. Deionized water was produced by the ultrapure water purification system MicroPure UV (TKA Wasseraufbereitungssysteme GmbH, Niederelbert, Germany) equipped with a 0.2 µm filter; therefore, no further sterilization was needed. After preparation, all liposomal formulations were placed in a refrigerator (+4 °C) for 24 h for sedimentation of large aggregates. For further analysis and treatment of MGD and DES, superficial fractions of liposomal formulations (~80%) were aspirated and vortexed, subdivided into aliquots of 0.5 mL, and kept in the dark at 4 °C.

### 2.3. Mean Particle Size and Zeta Potential

A hydrodynamic diameter and zeta potential were determined by particle size and zeta potential analyzer Zeta Plus PALS (Brookhaven Inc., Suffolk County, NY, USA). Studies were performed in triplicate on freshly prepared samples diluted in the ratio of 1:199 (*v*/*v*) in deionized water at room temperature (25 °C). Additional stability studies were performed following the same protocol after 1, 6, 11, and 15 days of storage at 4 °C in the dark.

### 2.4. Determination of CycA by HPLC

In this study, cyclosporine A was quantified using an adapted and validated isocratic HPLC method [26]. Briefly, the samples were transferred to sample vials and injected by an autosampling injector (Gilson 234, Gilson, Villiers le Bel, France) into a 5 μm SUPELCOSIL™ LC-18 column (4.6 mm × 250 mm) (SUPELCO, St. Louis, MI, USA) protected by a 5 μm SUPELCOSIL™ LC-18 Supelguard™ (20 mm × 4 mm) (SUPELCO, St. Louis, USA) pre-column guard cartridge. In the injector rack, the temperature of samples was kept stable at 30 °C by a temperature regulator (model 832, Gilson, Villiers le Bel, France). The mobile phase of the isocratic method consisted of ROTISOLV^®^HPLC gradient-grade acetonitrile and water (70:30 *v*/*v*) purchased from ROTH (Carl Roth, Karlsruhe, Germany). Complete separation was achieved in 30 min by injecting 20 µL of samples and keeping the column oven (CTO-10A, Shimadzu, Kyoto, Japan) temperature at 50 °C. A low-pressure gradient pump system (P580A LPG, Dionex, Germany) with an integrated degasser was used to generate a flow rate of 0.7 mL/min. The column effluent was monitored at 210 nm by a UV detector (Spectra 200, Spectra-Physics, Milpitas, CA, USA) connected to the chromatography interface (UCI-100, Dionex, Germany) for analog to digital signal conversion. All the chromatographic separations were carried out at ambient temperature. Chromeleon software (version 6.50, Dionex) was used for data acquisition and calculations of the peak areas. Further data analysis was performed using SigmaPlot version 12.5 (Systat Software, Inc., Chicago, IL, USA). Calibration curves for cyclosporine A for each time point were made by plotting the peak area against the concentration (range 0.005–0.1 mg/mL, Supplementary Material, Figure S1). The concentrations of cyclosporine A were found by relating the corresponding peak areas to the obtained regression line. In order to measure the cyclosporine content in liposomal formulation, first Triton X-100 was mixed with a small amount of liposomal formulation to disrupt the liposomes. After a few minutes, an appropriate amount of water was added to maintain the dilution ratio as it is described in calibration curve measurements.

### 2.5. Determination of Curcumin by UV-VIS Spectroscopy

Determination of curcumin content by UV-VIS spectroscopy was adapted by a validated method [27]. Stock solution of curcumin was prepared in ethanol (0.2 mg/mL) and diluted 40 times with water/triton X-100 (11:2 *v*/*v*) mixture. Optical density was registered by UV-visible absorption spectrometer Varian Carry 50 (Varian Inc., Belrose, NSW, Australia), and values at 427 nm were plotted as a function of concentration. The standard calibration curve of curcumin was obtained by measuring the absorbance of curcumin solutions in the concentration range of 1–7 μg/mL (Supplementary Material, Figure S2).

In order to measure the curcumin content in liposomal formulation, first Triton X-100 was mixed with a small amount of liposomal formulation to disrupt the liposomes. After a few minutes, an appropriate amount of water was added to maintain the water/Triton X-100 ratio as it was described previously in calibration curve measurements. Samples were analyzed by UV-visible absorption spectrometer Varian Carry 50.

### 2.6. Encapsulation Efficiency by Protamine Aggregation

Protamine-induced aggregation was proposed as an economic and simple method to evaluate encapsulation efficiency [28]. The above-mentioned method was modified according to our application (negatively charged liposomes) and used in this study. Briefly, 100 µL of liposomal suspension (lipid concentration of 25 mg/mL) was thoroughly mixed with 100 µL of protamine solution (10 mg/mL) and allowed to stand for 3 min at room temperature. To this mixture, 3 mL of 0.9% NaCl was added. The tubes were centrifuged at 2000× *g* for 20 min. The top 2 mL of supernatant was aspirated and analyzed for an unentrapped compound by HPLC or UV-visible absorption spectroscopy. The total amount of compound within 100 µL liposomal suspension was evaluated by HPLC (for cyclosporine A) or UV-visible absorption spectroscopy (for curcumin) as described in previous methods. Encapsulation efficiency % (EE(%)) was calculated by applying (1) formula:

(1) $$EE\left(\%\right)=\frac{\mathrm{Total}\;\mathrm{mass}\;\mathrm{of}\;\mathrm{compound}-\mathrm{Unentrapped}\;\mathrm{mass}\;\mathrm{of}\;\mathrm{compound}}{\mathrm{Total}\;\mathrm{mass}\;\mathrm{of}\;\mathrm{compound}}\cdot 100\%$$

### 2.7. Microscopy Analysis of Liposomes

On day 15 after liposome preparation, 10 µL of a sample (Lipo@Cur or Lipo@CycA) was placed under confocal microscope Nikon Eclipse C1si (Nikon Instruments, Inc., Tokyo, Japan) for examination. Oil-immersion objective was used with a magnification of 60× (Plan Apo 60×/1.40). Transmission confocal microscopy images were captured using a 488 nm laser as a light source and transmission detector unit. Images were analyzed using EZ-C1 software (version 3.90).

### 2.8. Animal Model and Ethical Considerations

All animals were treated according to the Association for Research in Vision and Ophthalmology (ARVO) Statement for the Use of Animals in Ophthalmic and Vision Research and the EC Directive 86/609/EEC for animal experiments using protocols approved and monitored by the State Food and Veterinary Service of Lithuania (animal ethical license number G2-213). New Zealand white rabbits, males, 10–12 weeks old, 2.0–2.5 kg, were purchased from the Innovative Medicine Center, Vilnius.

All rabbits were housed at a constant temperature of 22 ± 1 °C and a humidity of 55% ± 10% in a light-controlled environment (lights on from 7 a.m. to 7 p.m.) with ad libitum access to food and water. They were housed in their cages individually.

### 2.9. DES and MGD Induction, Treatment

The animal models of DES and MGD were established as follows: under general anesthesia (Ketamin 40 mg/kg + Xylasin 5 mg/kg), complete Freund’s adjuvant (CFA) containing killed Mycobacterium lyophilized cells (Sigma-Aldrich, USA) (each 30 µL) was injected into the nasal, central, and temporal upper eyelid margin of the right eye [4]. Saline was injected into the left eye as a control into the respective places of the left upper eyelid. For 21 days, rabbits were housed normally until DES and MGD developed.

**Figure 2:**
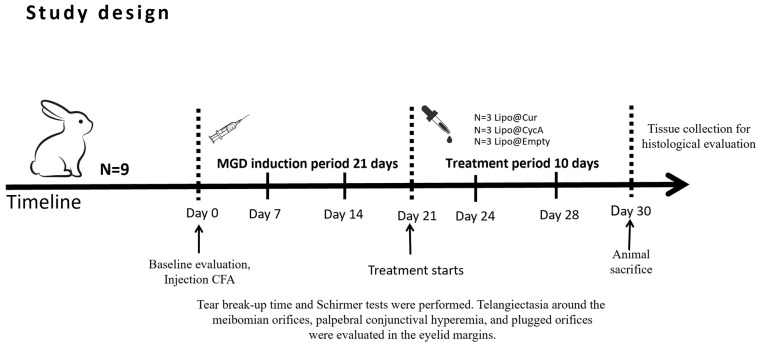
Study design of meibomian gland dysfunction (MGD) and dry eye syndrome (DES) induction and treatment with curcumin-loaded liposomes (Lipo@Cur), cyclosporine A-loaded liposomes (Lipo@CycA), and empty liposomes (Empty@Lipo) eye drops.

Treatment started on day 21 (Figure 2). Rabbits were randomly divided into five groups: curcumin-loaded liposomes (Lipo@Cur, N = 3), cyclosporine A-loaded liposomes (Lipo@CycA, N = 3), empty liposomes group (Empty@Lipo, N = 3), and the two control groups (artificial tear)—healthy and inflamed. All of them were administered with 30 μL drops twice a day (8 a.m. and 8 p.m.). Tear production (Schirmer test), TBUT, blockage of meibomian gland orifices, and hyperemia of conjunctiva and eyelid margin were observed at day 0, day 7, 14, 21, 24, 28, and day 30.

### 2.10. Schirmer Test

Tear production was measured using Schirmer tear test ophthalmic strips (Optitech Eyecare, Prayagraj, India). The test was performed on days 0, 7, 14, 21, 24, 28, and 30. Tear production was measured at baseline before CFA induction. Rabbits were restrained with cloth for immobilization. A strip of Schirmer paper was placed at the site of the conjunctival sac around the junction of the middle and outer thirds of the lower eyelid. The wetted length, in millimeters, was measured after 1 min. Every step was repeated 3 times. Paper strips that did not get wet were ignored. The average result was recorded as the final result.

Due to high diversity in rabbits’ tear production (see Supplementary Material, Table S1), relative tear production values (%) were used to compare tear production changes in each individual rabbit. The actual length value of the wetted strip was divided by a starting value (Day 0) and multiplied by 100 %. See Formula (2) for calculation description.

(2) $$\mathrm{Tear}\;\mathrm{Production}\left(\%\right)=\frac{\mathrm{Lx}}{L0}\cdot 100\%$$

*Lx*—Actual length (mm) of wetted ophthalmic strip at a given time point, from day 0 till day 30.
*L0*—Length (mm) of a wetted ophthalmic strip at day 0 (before experiments).

### 2.11. TBUT Test

TBUT was used to assess evaporative DES. Tests were performed on days 0, 7, 14, 21, 24, 28, and 30 under anesthesia (Ketamin 40 mg/kg + Xylasin 5 mg/kg). The fluorescein sodium ophthalmic strips U.S.P. (Optitech eyecare, Prayagraj, India) were instilled into the eye. Then, the tear film was observed under a broad beam of cobalt blue illumination. The TBUT was recorded as the number of seconds that elapse between the last blink and the appearance of the first dry spot in the tear film.

We converted the TBUT results into percentage changes because individual rabbits displayed variability in their baseline, healthy TBUT measurements. By expressing the changes as percentages, we aimed to better reflect the relative impact of the treatments on each group. The formula used was as follows:

(3) $$\mathrm{TBUT}\;\mathrm{percentage}\;\mathrm{change}\left(\%\right)=\frac{\mathrm{TBUT}\;\mathrm{on}\;\mathrm{DayX}}{\mathrm{TBUT}\;\mathrm{on}\;\mathrm{Day}21}\cdot 100\%$$

where ‘DayX’ refers to subsequent measurement days (e.g., Day 24, Day 28, or Day 30). This approach allowed us to demonstrate the increase or decrease in TBUT for each treatment group, using Day 21 as the baseline for comparison within each group.

### 2.12. Rabbit Sacrifice and Histopathology Specimen Collection

At the end of the study on the 30th day, the rabbits were euthanized by overdose of pentobarbital injection (Exagon 400 mg/mL, VetViva Richter GmbH, Wels, Austria) administered intravenously into the auricular vein. The anatomical structure of the cornea and conjunctiva was observed by H and E staining and periodic acid–Schiff. Ocular tissues—eyelids, cornea, and conjunctiva—were dissected after euthanasia on day 30 after CFA injection. All tissues were fixed overnight in 4% paraformaldehyde saline. Then, tissues were embedded in the FFPI blocks, cut into 8 µm layers, and stained with hematoxylin–eosin (HE), periodic acid–Schiff (PAS), and Alcian Blue (AB). The histology slides were prepared and evaluated by the National Center of Pathology. Slides were magnified 10x, scanned, and pictured using Kern Optics Digital Microscope OBN 135 (KERN & SOHN GmbH, Balingen, Germany) and Kern optics tablet cam ODC 241 (KERN & SOHN GmbH, Balingen, Germany).

### 2.13. Clinical Evaluation of the Eyelids

Telangiectasia around the meibomian orifices, palpebral conjunctival hyperemia, and plugged orifices were observed in the eyelid margins. Changes were assessed under a slit lamp (LED slit lamp SL9900 5xD, CSO srl, Scandicci FI, Italia) and imaged using a digital camera (Leica DMC6200, Leica Microsystems, Wetzlar, Germany). All images were obtained using the same camera with the same settings. Assessment was performed in a 0–3 point system: 0 points were given when no redness or no blocked orifices were observed; 1 point was given for light redness and up to 4 detected blocked orifices; 2 points—average severity of telangiectasia around the orifices, hyperemia, and 4 to 7 blocked orifices; 3 points—for severe telangiectasia around the orifices, hyperemia, and >7 blocked orifices. The eyelid margin was divided into 3 zones: temporal, central, and nasal, and each zone was assessed separately. The final score was summed from all 3 zones. The highest score would be 9, and the lowest would be 0 points. Blocked meibomian orifices, telangiectasia, and hyperemia were evaluated separately. The examination was performed on days 0, 7, 21, 24, 28, and 30 under anesthesia (Ketamin 40 mg/kg + Xylasin 5 mg/kg).

### 2.14. Statistical Analysis

The statistical analysis was performed using graph pad analysis software (Prism 10.3.0). Two-Way ANOVA with Dunnett’s multiple comparison test was performed to determine statistical significance for the Schirmer test, TBUT, telangiectasia score, and plugged orifice score between untreated control group and Lipo@Cur, Lipo@CycA, and Empty@Lipo compound-treated groups. The data are expressed as mean ± Standard Error of the Mean (SEM). Differences are considered statistically significant at *p* < 0.05.

## 3. Results

### 3.1. Characterization and Stability of Liposomal Formulations

The preparation procedure of Lipo@Cur and Lipo@CycA involved heating and sedimentation steps; therefore, the concentration of the active anti-inflammatory compounds was measured immediately after eye drop production. For the Lipo@Cur sample, the concentration of curcumin was almost the same as theoretical, confirming the successful production procedure (Table 3). During the production of Lipo@CycA, 15% of the active compound was lost; therefore, the concentration of cyclosporine A in eye drops was 0.85 mg/mL (0.085%). Encapsulation efficiencies of cyclosporine A and curcumin were 64% and 93%, respectively (Table 3).

**Figure 3:**
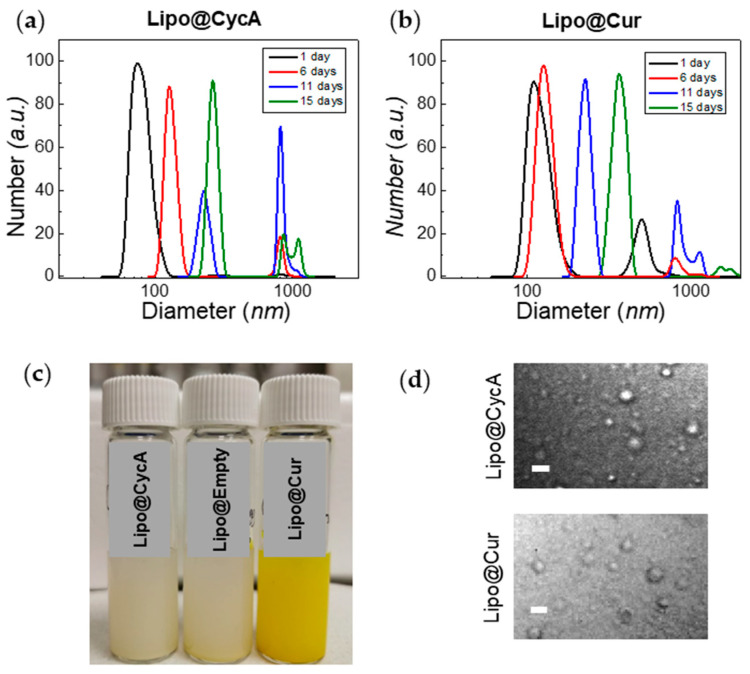
Changes in hydrodynamic diameter of Lipo@CycA (**a**) and Lipo@Cur (**b**) particles over time. The color and opacity of Lipo@CycA, Lipo@Cur, and Lipo@Empty formulations can be seen in Section (**c**). At day 15, the fraction of small-sized liposomes was discernible and captured with confocal microscopy transmission detector unit (**d**), magnification 60×, oil-immersion objective. Scale bar = 4 µm.

The zeta potential of liposomes was about −7 eV for Lipo@Empty and Lipo@CycA, and −9 eV for Lipo@Cur (Table 3). The polydispersity index (PDI) of Lipo@CycA, Lipo@Cur, and Lipo@Empty was similar between formulations, ranging from 0.34 to 0.36, indicating the heterogeneity of a sample in terms of particle size. The dynamic light scattering measurements correlated well with the PDI and revealed a two-component distribution for both Lipo@CycA and Lipo@Cur (Figure 3a,b). The average hydrodynamic diameter was ~90 nm for Lipo@CycA and ~150 nm for Lipo@Cur. The large-sized distribution had an average of ~1000 nm for both formulations and comprised less than 5% of the total distribution.

The treatment of DES and MGD lasted 10 days; therefore, the stability of the liposomal formulations was tested for at least 10 days. Neither the zeta potential nor the encapsulation efficiency of all liposomal formulations changed over 15 days. The hydrodynamic diameter had changed over time, indicating the agglomeration and merging of liposomes (Figure 3a,b). On day 15, the fraction of the large-sized liposomes reached 14% and was detected by confocal microscopy with a transmission detector unit with oil-immersion objective, magnification 60× (Figure 3d). Round-shaped particles could be seen in microscopy images, thus proving the presence of small-sized liposomes in Lipo@CycA and Lipo@Cur eye drops. The concentration of active compounds, cyclosporine A and curcumin, was followed for 10 days. No changes were detected in the cyclosporine A concentration, while the concentration of curcumin slightly decreased from 1.05 mg/mL to 0.99 mg/mL (by ~6%) at day 10.

### 3.2. Treatment of DES and MGD In Vivo

#### 3.2.1. Tear Film Stability and Tear Volume After MGD Induction and Treatment

**Figure 4:**
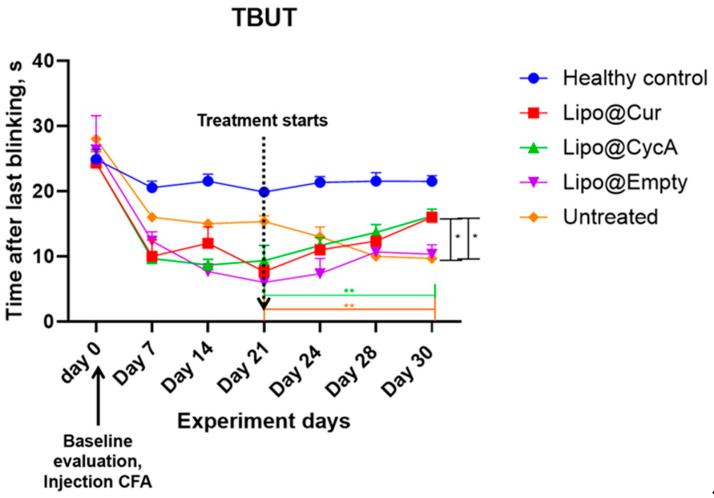
Seven days after injection of complete Freund’s adjuvant (CFA), tear break-up time (TBUT) reduced around 50%, proving development of DES model. After the start of the treatment, TBUT improved in Lipo groups. Ordinary two-way ANOVA test revealed main significant effects of treatment, time point, and interaction between both factors. Subsequent Dunnett’s multiple comparison test analysis showed a significant difference in TBUT in the treatment groups at the last day of treatment compared to the healthy control group. * *p* < 0.05; ** *p* < 0.005.

The results presented in Figure 4 indicate a significant reduction in tear break-up time (TBUT) by approximately 50% seven days after the injection of complete Freund’s adjuvant (CFA), confirming the successful induction of the DES model. Following the initiation of treatment, TBUT improved in the groups receiving Lipo-based treatments. A two-way ANOVA analysis revealed significant main effects of both the treatment and time point, as well as a significant interaction between these factors. Further analysis using Dunnett’s multiple comparison test demonstrated a significant improvement in TBUT for the treatment groups compared to the healthy control group on the final day of treatment (* *p* < 0.05; ** *p* < 0.005). This indicates the efficacy of the Lipo treatments in improving TBUT over the treatment period.

Additionally, Table 4 helps to better understand the results of Figure 4. TBUT across all groups was calculated as percentages from their baseline values; therefore, on Day 21 (the start of the treatment), all groups began with 0% change. By Day 30, the Lipo@Cur group reached a 109% improvement, Lipo@CycA achieved 73%, Lipo@empty saw 83%, while the untreated group experienced a continued decline to −37%. These data highlight the superior improvement in TBUT in the Lipo-treated groups, particularly with Lipo@Cur, compared to both the untreated and healthy control groups.

**Figure 5:**
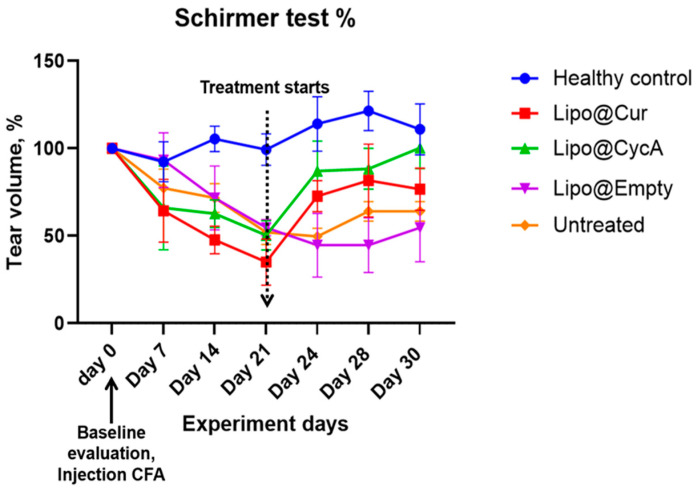
Schirmer test results of tear production alteration during experiment period in percentages. The average tear production in all groups during the experiment period decreased after the injection of CFA in all groups at warehouse ratings until day 21. From that day, Lipo@Cur and Lipo@CycA showed quite a steep increase in tear production when compared with other groups, which still showed a slight decrease in tear production. Two-way repeated measures ANOVA test did not reveal a significant effect on tear production among all treatment groups when compared with untreated control.

The results presented in Figure 5 illustrate the changes in tear production, measured by the Schirmer test, during the experiment period. Tear production in all the groups decreased following the injection of complete Freund’s adjuvant (CFA), implying the development of DES. This decline persisted in all groups up to Day 21, at which point a marked difference emerged in the treatment groups. Both Lipo@Cur and Lipo@CycA groups showed a significant increase in tear production compared to the untreated and other control groups. The untreated group continued to exhibit a slight decrease in tear production through all days of the experiment.

Despite the observed trend, a two-way repeated measures ANOVA did not reveal a statistically significant effect on tear production between the treatment groups when compared with the untreated control. This suggests that while Lipo@Cur and Lipo@CycA may have induced improvements in tear production, these changes were not sufficient to be statistically different across the experiment timeframe.

Table 5 shows the percentage changes in tear volume across all groups during the experiment. At Day 21 (0 days), tear volume was normalized to 0% for all groups. By Day 24 (3 days), the Lipo@Cur group showed a significant increase in tear volume (115%), followed by Lipo@CycA (73%), while the Lipo@empty group showed a decrease (−18%) and the untreated group had a slight reduction (−4%). On Day 28 (7 days), the tear production continued to rise in the Lipo@Cur group (141%) and remained elevated in the Lipo@CycA group (75%), while Lipo@empty still showed a decline (−18%) and the untreated group saw a modest increase (23%). By Day 30 (9 days), tear volume remained high in the Lipo@Cur (119%) and Lipo@CycA (99%) groups, whereas Lipo@empty showed no change (0%) and the untreated group had a small increase (23%). Overall, the Lipo@Cur and Lipo@CycA groups demonstrated significant improvements in tear production, while the Lipo@empty and untreated groups showed minimal or negative changes.

#### 3.2.2. Clinical Evaluation of MGD Symptoms

**Figure 6:**
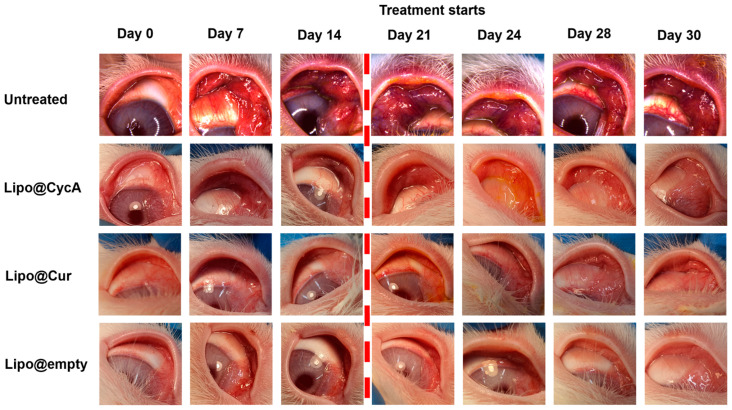
Visual representation of the changes in the eyelids and conjunctiva across different treatment groups. The untreated group images appear more reddish, indicating significant inflammation and a lack of improvement. In contrast, the liposomal treatment groups (Lipo@Cur, Lipo@CycA, and Lipo@Empty) exhibit less pronounced redness, demonstrating the effectiveness of these treatments in reducing visible symptoms of inflammation. The purpose of this figure is to provide a qualitative introduction to the detailed quantitative analysis presented in Figure 7, where specific clinical symptoms, such as plugged orifices and telangiectasia scores, are statistically analyzed.

**Figure 7:**
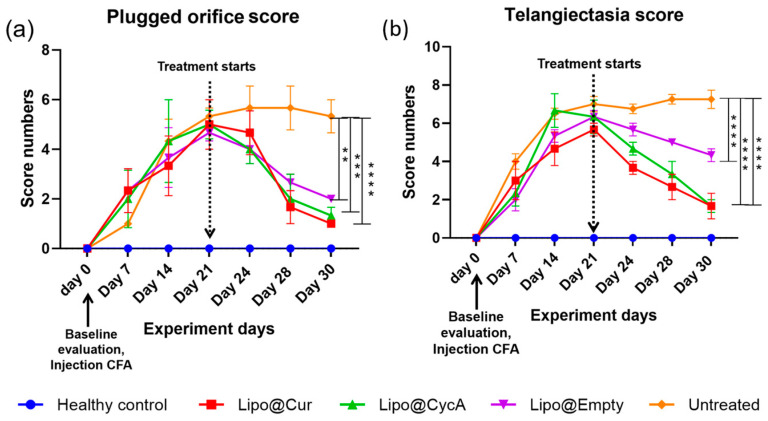
Plugged orifice (**a**) and telangiectasia (**b**) scores. After the injection of CFA, telangiectasia and palpebral conjunctiva hyperemia as signs of inflammation were observed in all groups except healthy control. Once the treatment started, the incidence of inflammation gradually decreased, except for the untreated group. Ordinary two-way ANOVA test revealed significant main effects of treatment, time point, and interaction between both factors. Subsequent Dunnett’s multiple comparison test analysis showed a significant difference in plugged orifice scores in the treatment groups on the last day of treatment compared to the untreated group. Differences are considered to be statistically significant at *p* < 0.05 level, ** *p* < 0.0032; *** *p* < 0.0003; **** *p* < 0.0001.

Figure 6 visually illustrates the changes in eyelid and conjunctiva appearance across the different treatment groups. Before the treatment began on Day 21, all groups exhibited increasing signs of inflammation, characterized by a progressive redness in the eyelids and conjunctiva (left side pictures). The untreated group showed pronounced redness, indicating significant inflammation and a lack of improvement. In contrast, the liposomal treatment groups (Lipo@Cur, Lipo@CycA, and Lipo@Empty) exhibited a reduction in redness after treatment, suggesting the effectiveness of these interventions in alleviating visible symptoms of inflammation. This figure provides a qualitative introduction, highlighting the visual differences between groups, and sets the stage for a more detailed quantitative analysis in Figure 7, where specific clinical symptoms, such as plugged orifices and telangiectasia scores, are statistically evaluated.

Figure 7 presents the scores for plugged orifice (Figure 7a) and telangiectasia (Figure 7b). Following the injection of complete Freund’s adjuvant (CFA), signs of inflammation, including telangiectasia and palpebral conjunctiva hyperemia, were observed in all groups except the healthy control group. Once the treatment began, the inflammation gradually decreased in all the treatment groups, while the untreated group continued to show persistent inflammation. An ordinary two-way ANOVA test revealed significant main effects of the treatment, the time point, and the interaction between these two factors. Subsequent analysis using Dunnett’s multiple comparison test indicated a significant reduction in the plugged orifice scores in the treatment groups on the last day of treatment compared to the untreated group. Differences were considered statistically significant at *p* < 0.05 level, with further significance levels noted as ** *p* < 0.0032, *** *p* < 0.0003, and **** *p* < 0.0001.

#### 3.2.3. Histopathology Specimen Evaluation

**Figure 8:**
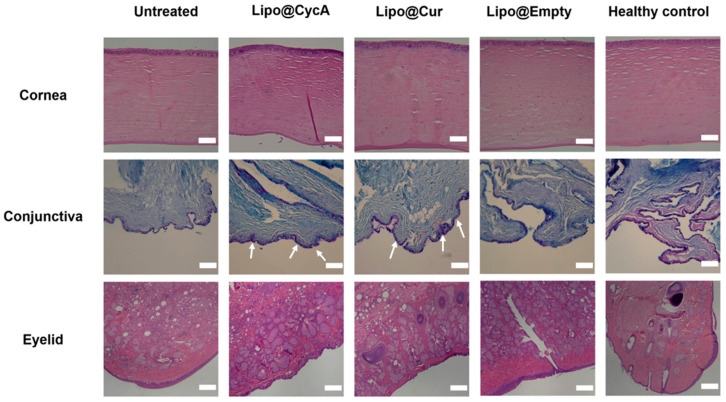
Microscopic alterations in the cornea, conjunctiva, and eyelids across all researched groups. Histological samples of cornea and eyelid were stained with hematoxylin–eosin (HE), and conjunctiva with Alcian Blue (AB) and Periodic Acid–Schiff (PAS). The white arrows show goblet cells in conjunctiva. Cornea histomorphology showed epithelial thinning mainly in the untreated group. Diffuse histiocytic granulomas in the eyelid tissues of CFA-injected groups were consistent with conjunctival inflammation. The scale bar is 200 µm.

On the last day of the experiment (day 30), ocular tissues, including the eyelids, cornea, and conjunctiva, were removed and preserved in formalin for histopathological evaluation. This analysis can provide valuable insights into the underlying causes of dry eye syndrome (DES) and meibomian gland dysfunction (MGD) by examining tissue samples for any abnormalities or changes at a cellular level. Figure 8 illustrates the microscopic alterations in the cornea, conjunctiva, and eyelids across all the researched groups. Histological samples of the cornea and eyelid were stained using hematoxylin–eosin (HE), while the conjunctiva was stained with Alcian Blue (AB) and Periodic Acid–Schiff (PAS). The corneal histomorphology revealed epithelial thinning, particularly in the untreated group. Additionally, diffuse histiocytic granulomas were observed in the eyelid tissues of the CFA-injected groups, indicating conjunctival inflammation. The scale bar represents 200 µm. In the untreated group, focal lymphocytic subepithelial infiltration was observed, indicating inflammation and poor tear film quality. The Lipo@CycA and Lipo@Cur groups had goblet cells in all samples (Figure 8, white arrows). The Lipo@Empty group showed goblet cells, but less than Lipo@CycA and Lipo@Cur, indicating minimal therapeutic benefit from empty liposomes. The healthy control group exhibited no histiocytic granuloma formation, only goblet cells, indicating normal histology without significant inflammatory response. The presence of diffuse histiocytic granulomas in the eyelid tissues of the treated groups (Lipo@Cur, Lipo@CycA, and Lipo@Empty) was consistent with the inflammatory response observed in the conjunctiva. The eyelids from the untreated group showed a significant prevalence of diffuse histiocytic granulomas with a limited presence of eosinophils and plasma cells in the deep dermis.

## 4. Discussion

Lipo@CycA, Lipo@Cur, and Lipo@Empty were assembled using a thin-film hydration method. It is an old method, but many scientists still use this method as a reliable and reproducible way for the lab-scale production of quality liposomes [25]. Common eye drop medications with CycA contain 0.05 % to 0.1 % CycA; therefore, our formulation fits the therapeutic dose proven suitable by the U.S. Food and Drug Administration (FDA) for long-term usage [29]. The characterization of liposomes revealed that most of the active compound was encapsulated inside the liposomes (>65%). Both curcumin and cyclosporine A are poorly soluble in water; therefore, solubilizing excipients, such as PLGA, TPGS, or phospholipids, must be used to form aqueous emulsions [30,31]. We chose to use a mixture of natural phospholipids to mimic the biological environment of the natural tear film (Figure 1). The liposomes formed were rather small, with an average hydrodynamic diameter of ~90 nm for Lipo@CycA and ~150 nm for Lipo@Cur, but during 10 days of storage, a fraction of large-sized liposomes (~1000–2000 nm) had formed via fusion of small liposomes (Figure 3). FDA-approved liposomes vary in diameter from 20 nm to 30.000 nm [32]. Small-sized liposomes (up to 50 nm) are usually required for deeper tissue penetration or longer circulation in the bloodstream [14]. When slow-release kinetics are needed and for immobilization, for instance in pain management, large-sized liposomes or liposome micro-clusters are produced [15]. Tear film is present on the surface of corneal epithelium; therefore, the replenishment of the DES-affected tear film should also target the surface of corneal epithelium (Figure 1a). A longer presence of eye drops on the surface of the eyes could lead to better hydration and evaporation prevention. Marta Vicario-de-la-Torre et al. have developed eye drop formulations containing 100 to 1000 nm liposomes intended for a longer presence at the surface of the cornea and relieving the signs and symptoms of DES [33].

The main active component of Lipo@CycA was stable during 10 days of storage, while Lipo@Cur formulation had a decline in its active compound by 6%. In other studies, curcumin, encapsulated in liposomes, degraded by 40 % within 8 h at physiological pH [34]. Chitosan was suggested as a stabilizing agent. Thus, we can conclude that our biomimetic curcumin-loaded liposomes showed exceptional stability within 10 days without additional excipients. But, for long-term storage, some excipients might be needed for better stability of curcumin in a Lipo@Cur formulation.

The main goal of this study was to assess the clinical effect of Lipo@Cur and Lipo@CycA on rabbit models with induced MGD and DES. By investigating the efficacy of these treatments, we first evaluated the clinical parameters, such as tear film stability (TBUT) and tear volume (Schirmer). TBUT indicates the stability of the tear film, which is disrupted in DES. Generally, TBUT of >10 s is thought to be normal [35]. Short TBUT is associated with unstable tear film and severe DES. The Schirmer test is used to determine whether the eye produces enough tears to keep it moist. The Schirmer test measurement takes 5 min, and the results of 0–5 mm indicate severe dry eyes, 5–10 mm—moderately dry eyes, and >10 mm—normal tear function [36].

After injection of CFA into the right eyelids, TBUT reduced by around 50% in all CFA-treated groups, similar to previous reports [36]. The shortening of TBUT is associated with the development of DES. The left eye of rabbits was used as a healthy control group, i.e., without an induced DES; therefore, almost no change in TBUT results was observed throughout the whole period of experiment (Figure 4). There was an improvement in the TBUT of the Lipo@Cur, Lipo@CycA, and Empty@Lipo groups soon after starting the treatment (Table 4). The results suggest that Lipo@Cur treatment has the most beneficial impact on increasing TBUT, followed by Lipo@CycA, while the untreated group displays a consistent decrease in tear film stability over the observation period. Notably, empty liposomes, Lipo@Empty, demonstrate an improved clinical efficacy of dry eye symptoms and tear film stability, which is essential to reduce the evaporation of the tear film lipid layer.

The injection of CFA into eyelids of experimental rabbits caused meibomian gland inflammation and led to MGD [37]. When meibomian glands are affected, the lipid layer of the tear film is also impaired, leading to evaporative DES. Tear volume results suggest that the Lipo@Cur and Lipo@CycA treatments hold promise in comparison to both the healthy control and the untreated group, while Lipo@Empty’s outcomes are less predictable. It can be assumed that liposomes do not have an anti-inflammatory effect; therefore, loading of anti-inflammatory compounds (cyclosporine A or curcumin) in liposomes is an important strategy to control DES and MGD symptoms. It is worth mentioning that at day 21, left healthy eyes (without CFA) of rabbits showed rising Schirmer test results (Table 5, control group) and this may be caused by reflex tearing [36]. When one eye experiences dryness or irritation, it can send signals through nerve pathways to stimulate tear production in both eyes. This reflexive response aims to protect and lubricate both eyes when one eye is compromised. As a result, tear production may be increased not only in the MGD and DES right eye of rabbits, but also in the left healthy eye.

Over time, a range of therapies have been suggested for managing dry eye, with anti-inflammatory eye drops being one option. Recently, various nanomaterials and liposomal carriers have been suggested for the management of dry eyes [10]. In our study, following the creation of biomimetic liposomes containing curcumin, cyclosporine A, and just phospholipids, they were assessed for clinical effectiveness using a rabbit model with MGD and DES induced by CFA injection. Our findings suggest that both empty liposomes and curcumin loaded liposomes as nanotechnology-based eye drops positively impact the ocular surface after induced DES and improve tear film quality. Cyclosporine A is a common drug utilized in treating DES [38]. The clinical efficacy of cyclosporine A has been proved in PLGA nanoparticles, but not liposomes. We suggest that the inflammation caused by dry eyes of the ocular surface can be alleviated by incorporating natural substances, like phospholipids and curcumin, which are known for their anti-inflammatory properties. Studies have shown that curcumin can suppress the production of pro-inflammatory cytokines, like interleukin (IL)-4 and IL-5, in the conjunctiva of mice sensitized with ovalbumin [39]. Another study showed the effectiveness of curcumin tablets as an alternative to tear substitutes for managing dry eye disease. With the use of curcumin tablets, the frequency of artificial tear use was reduced compared to a placebo group [40]. To our knowledge, curcumin has never been used in a formulation of liposomal eye drops for managing dry eye symptoms. Therefore, our study contributes to the new knowledge regarding the action of curcumin once applied topically.

In a study conducted in Germany, it was found that the use of liposome drops alone led to improvements in the Schirmer test results for dry eye treatment [41]. Cyclosporine as an effective treatment has been proven in managing dry eye disease and has shown positive outcomes in improving the Schirmer test results. After a 6-month treatment with topical cyclosporine A, there was a statistically significant increase in the median Schirmer score from 3.00 mm to 4.00 mm [42]. However, limited research exists regarding the efficacy of cyclosporine incorporated into liposomes in treating this condition. Our findings demonstrate that cyclosporine A, by reducing inflammation, increases tear production and directly influences the outcomes of the Schirmer test: after 9 days of treatment, the production of tears increased by 99%. There are successful reports about the delivery of phospholipid liposomes to the tear film by applying a liposomal spray to the surface of the closed eyes. Results showed improvement in ocular comfort, lipid layer thickness, and tear film stability in healthy patients versus a control group where saline spray was used [43]. Our findings highlight the prospect of curcumin- and cyclosporine-loaded liposomes in enhancing therapeutic efficacy in DES, revealing the importance of formulation choice in achieving the best treatment outcomes.

The clinical characteristics of MGD can be internal or external. Internal features pertain to issues specifically within the meibomian glands or the surrounding lid tissues, such as blocked openings, obstructions in the ducts, enlargement or shrinkage of the glands, and changes in the quality of secretions. External features refer to secondary changes that occur due to MGD but are also observed in other types of ocular surface diseases, including redness and visible blood vessels along the edge of the eyelids. Figure 6 presents visually assessed telangiectasia, conjunctival hyperemia, and plugged orifice changes among all study groups. Following the administration of CFA, prominent signs of eyelid hyperemia and thickening of the lid rim were consistently observed in the rabbits’ eyes throughout the study period, showing minimal to exacerbated changes. In all the treated groups, positive changes were observed when comparing the initial and final days of the experiment, demonstrating improvements in hyperemia and telangiectasia. Empty liposomes do not have such significant anti-inflammatory properties; so, they do not show a positive effect in our study. Dry eyes can be caused by inflammation, which can disrupt tear production and quality, causing dryness and discomfort. Thus, in the treatment of dry eyes, moisturizing is not enough; additional anti-inflammatory treatment is needed. The untreated group showed persistent and sometimes worsening signs of eyelid hyperemia and thickening throughout the study period (Figure 6). This suggests that without intervention, these symptoms may continue to worsen over time.

Furthermore, our study evaluated the scores of clinical signs associated with MGD and DES, including telangiectasia around the meibomian orifices, palpebral conjunctiva hyperemia, and blocked orifices (Figure 7a,b). Following the administration of CFA, score numbers started increasing up to day 21, reflecting the inflammatory response triggered by the experimental model. Treatment resulted in a decrease in the clinical signs of inflammation across all treatment groups, indicating the efficacy of the interventions in alleviating ocular surface inflammation associated with dry eye conditions.

Based on the literature data, cyclosporine A was found to reduce the blockage of meibomian gland orifices and improve the measurable indicators of MGD [44]. Other studies suggest that curcumin with potential anti-inflammatory effects may be useful in chronic DES, allergic conjunctivitis, and blepharitis [45].

The histopathology images of our research highlight the significant impact of DES on the structure and composition of the ocular surface (Figure 8). Untreated eyes are dry and exhibit notable changes in the corneal epithelium, including a reduction in thickness compared to both treated groups and healthy controls. This observation is consistent with existing literature that suggests a correlation between dry eyes and alterations in the corneal morphology [46]. The corneal epithelium comprises five to seven cell layers and serves as the outermost layer of the cornea. DES can lead to changes in the thickness of the corneal epithelium layer, with histological evaluation showing a thinner layer in the untreated group [47]. Goblet cells, which are specialized epithelial cells found in mucosal tissues, are specifically located within the conjunctival epithelium. Goblet cells play an important role in maintaining mucosal integrity and lubrication on the ocular surface, and their reduced density in the untreated group indicates a potential disruption in the ocular surface health [48]. The examination of untreated group eyelids histologically displayed an inflammatory pattern (Figure 8). This pattern included widespread histiocytic granulomas with few eosinophils and plasma cells. This indicates a transition to chronic inflammation without treatment, potentially impacting the progression of the disease. Overall, our results underscore the importance of timely intervention and treatment in managing MGD-associated DES to prevent structural and inflammatory changes in the ocular tissues.

## 5. Conclusions

In this comprehensive analysis of MGD and DES induction in rabbit models, our investigation revealed key insights into the therapeutic potential of biomimetic curcumin-loaded (Lipo@Cur) and cyclosporine A-Loaded (Lipo@CycA) liposomes in mitigating the manifestations of these conditions. Following CFA injection, a significant reduction in TBUT appeared, proving CFA as an appropriate choice for the modeling of MGD-associated tear film instability and DES. Notably, the treatment with the Lipo@Cur and Lipo@CycA formulations markedly improved TBUT, with Lipo@Cur exhibiting the most pronounced effect, suggesting its superior potential in MGD, restoring tear film stability, and mitigating dry eye symptoms. This is further substantiated by statistical analyses, including ordinary Two-way ANOVA and Dunnett’s multiple comparison test, which affirmed the significant impact of these treatments over the observation period. Clinical observations of telangiectasia, conjunctival hyperemia, and plugged meibomian orifices further elucidated the condition’s progression and the therapeutic interventions’ effectiveness. The untreated group displayed an exacerbation of these symptoms, highlighting the natural progression of DES and MGD in the absence of treatment. In contrast, all treated groups exhibited notable improvements, with Lipo@Cur and Lipo@CycA demonstrating significantly reduced inflammation and ocular surface changes. These outcomes not only validate the models used but also emphasize the critical role of specific treatments in addressing the underlying inflammatory mechanisms of DES and MGD.

In conclusion, our research substantiates the significant therapeutic potential of biomimetic Lipo@Cur and Lipo@CycA formulations in managing DES and MGD, highlighted by their ability to improve tear film stability, alleviate ocular surface inflammation, and prevent structural changes in ocular tissues. These findings not only contribute to the growing literature on the management of DES and MGD, but also highlight the need for timely and specific intervention to mitigate disease progression and the impact on ocular health. The efficacy of biomimetic liposomes addressing the complex manifestations of DES and MGD is a promising field for future research and treatment expansion using natural active ingredients, setting the stage for more effective and personalized treatment strategies in ophthalmic medicine.

## Figures and Tables

**Table 1:** Comparison between the composition of meibomian gland oil and phospholipids used in the production of liposomes.

Lipids	Phospholipid Composition of Liposomes mol (%)	Meibomian Gland Oil Composition mol (%) [23]
Phosphatidylcholine	33	40
Phosphatidylethanolamine	14	18
Phosphatidylinositol	8	5
Other	45	27

**Table 2:** Formulation nomenclature and composition.

Name	Lipids	Active Compound	Other Components
Lipo@CycA	Soy-PC 40%, 25 mg/mL	Cyclosporine A, 1 mg/mL	Deionized water, 0.154 M NaCl
Lipo@Cur	Soy-PC 40%, 25 mg/mL	Curcumin, 1 mg/mL	Deionized water, 0.154 M NaCl
Lipo@Empty	Soy-PC 40%, 25 mg/mL	-	Deionized water, 0.154 M NaCl

**Table 3:** Parameters of liposomal formulations immediately after production: Lipo@CycA, Lipo@Cur, and Lipo@Empty.

	Theoretical c	Measured c After Production	EE	ZETA, mV	PDI
Lipo@Empty	0 mg/mL	0 mg/mL	N/D	−7.3 ± 1.0	0.34
Lipo@CycA	1 mg/mL	0.85 mg/mL	64%	−7.1 ± 0.7	0.34
Lipo@Cur	1 mg/mL	1.05 mg/mL	93%	−9.4 ± 0.8	0.36

**Table 4:** Changes in TBUT in all groups were calculated as percentages.

Experiment Days (Treatment Days)	Healthy Control	Lipo@Cur	Lipo@CycA	Lipo@empty	Untreated
Day 21 (0 days)	0%	0%	0%	0%	0%
Day 24 (3 days)	8%	43%	25%	22%	−15%
Day 28 (7 days)	8%	61%	46%	78%	−35%
Day 30 (9 days)	9%	109%	73%	83%	−37%

**Table 5:** Changes in tear volume in all groups were calculated as percentages.

Experiment Days (Treatment Days)	Healthy Control	Lipo@Cur	Lipo@CycA	Lipo@empty	Untreated
Day 21 (0 days)	0%	0%	0%	0%	0%
Day 24 (3 days)	21%	115%	73%	−18%	−4%
Day 28 (7 days)	25%	141%	75%	−18%	23%
Day 30 (9 days)	12%	119%	99%	0%	23%

## Data Availability

Data are contained within the article.

## Supplementary Materials

The following supporting information can be downloaded at https://www.mdpi.com/article/10.3390/jcm13216436/s1: Figure S1: Representative chromatograms and the calibration curve of cyclosporine A; Figure S2: The absorption spectrum and the calibration of curcumin; and Table S1: Schirmer test results of each rabbit before CFA induction.
