# Supplementary material for: Biomimetic Curcumin-Loaded Liposomes for the Treatment of Dry Eyes and Meibomian Gland Dysfunction: An In Vivo Study
Source: J Clin Med. 2024 Oct 27;13(21):6436. doi: 10.3390/jcm13216436 (PMC11546689; doi:10.3390/jcm13216436)
Supplement: Supplementary file 1 [file jcm-13-06436-s001.zip › jcm-3219777-supplementary.pdf]

Supplementary Material

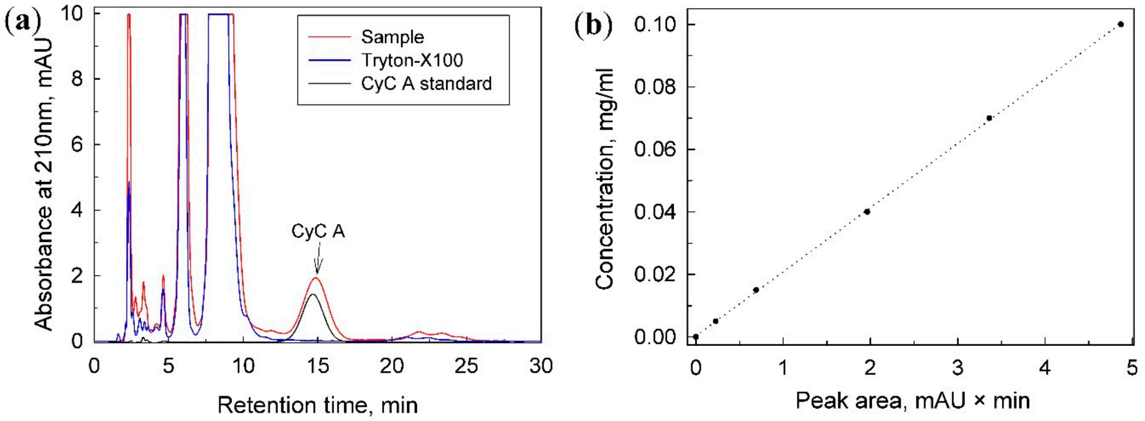

Figure S1. Representative chromatograms are present in section **(a)**. Triton X-100 was used for liposome disruption and CyC A release. CyC A standard and Triton X-100 working solutions were diluted in acetonitrile.The callibration curve demonstrates a linear detection system response to concentration over the range 0.005 - 0.1 mg/mL, R2= 0.9998 (**b**).

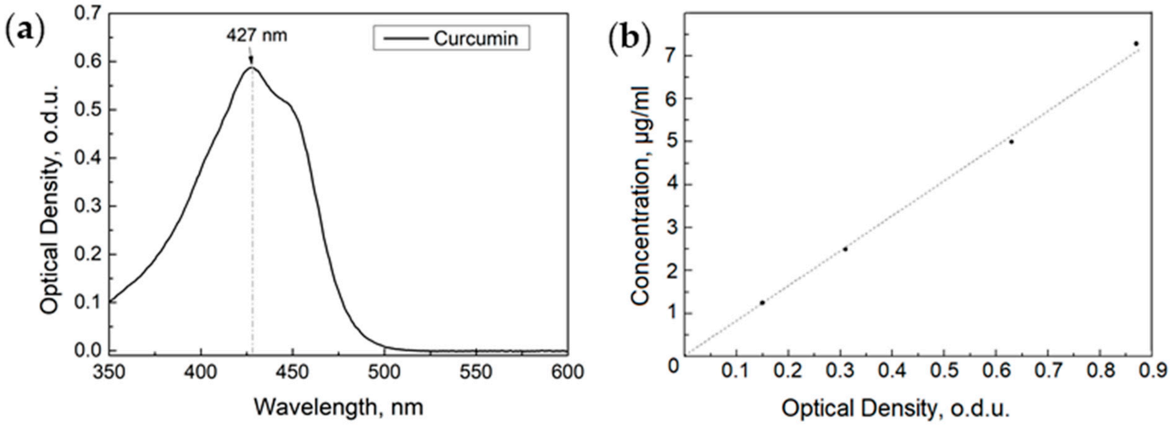

Figure S2. The absorption spectrum of curcumin, prepared in ethanol (0.2 mg/ml) and 40 times diluted in water/Triton X-100 (11:2 v/v) mixture (**a**). 4 different curcumin standard solutions were prepared within 1-7 µg/ml range and optical density values at 427 nm were plotted as a function of concentration (**b**). A linear dotted curve is a fitted mathematical curve. R2 = 0.9985.

Table S1. Schirmer test results of each rabbit before CFA induction expressed in wetted length of ophtalmic strip.

Rabbit Eyes	Rabbit 1	Rabbit 2	Rabbit 3	Rabbit 4	Rabbit 5	Rabbit 6	Rabbit 7	Rabbit 8	Rabbit 9
Right eye OD, mm	11.5	8.5	7	10	3	6	12	7	6
Left eye OS, mm	15.5	6.5	6	4.5	3	4.5	7.5	6.5	4.5
